# Diabetes care in remote Australia: the antenatal, postpartum and inter-pregnancy period

**DOI:** 10.1186/s12884-019-2562-6

**Published:** 2019-10-28

**Authors:** R. Kirkham, N. Trap-Jensen, J. A. Boyle, F. Barzi, E. L. M. Barr, C. Whitbread, P. Van Dokkum, M. Kirkwood, C. Connors, E. Moore, P. Zimmet, S. Corpus, A. J. Hanley, K. O’Dea, J. Oats, H. D. McIntyre, A. Brown, J. E. Shaw, L. Maple-Brown, On behalf of the NT Diabetes in Pregnancy Partnership

**Affiliations:** 1 Menzies School of Health Research, Darwin, Australia; 2 Monash Centre for Health Research and Implementation, School of Public Health and Preventive Medicine, Monash University, Melbourne, Australia; 3 Population Health Research, Baker Heart and Diabetes Institute, Melbourne, Australia; 4 Royal Darwin Hospital, Darwin, Australia; 5 Alice Springs Hospital, Alice Springs, Australia; 6 Population Health Research, Baker Heart and Diabetes Institute, Alice Springs, Australia; 7 Northern Territory Department of Health, Darwin, Australia; 8 Aboriginal Medical Services Alliance Northern Territory, Darwin, Australia; 9 Department of Diabetes, Central Clinical School, Monash University, Melbourne, Australia; 10 Danila Dilba Health Service, Darwin, Australia; 11 Department of Nutritional Sciences, Faculty of Medicine and the Dalla Lana School of Public Health, The University of Toronto, Toronto, Canada; 12 School of Health Sciences, University of South Australia, Adelaide, Australia; 13 Melbourne School of Population and Global Health, University of Melbourne, Melbourne, Australia; 14 Mater Medical Research Institute, University of Queensland, Brisbane, Australia; 15 South Australian Health and Medical Research Institute, Adelaide, Australia

**Keywords:** Diabetes in pregnancy, Postpartum, Antenatal, Healthcare services, Indigenous

## Abstract

**Background:**

Aboriginal and Torres Strait Islander women experience high rates of diabetes in pregnancy (DIP), contributing to health risks for mother and infant, and the intergenerational cycle of diabetes. By enhancing diabetes management during pregnancy, postpartum and the interval between pregnancies, the DIP Partnership aims to improve health outcomes and reduce risks early in the life-course. We describe a mixed methods formative study of health professional’s perspectives of antenatal and post-partum diabetes screening and management, including enablers and barriers to care.

**Methods:**

Health professionals involved in providing diabetes care in pregnancy, from a range of health services across the Northern Territory, completed the survey (*n* = 82) and/or took part in interviews and/or focus groups (*n* = 62).

**Results:**

Qualitative findings highlighted factors influencing the delivery of care as reported by health professionals, including: whose responsibility it is, access to care, the baby is the focus and pre-conception care. The main challenges were related to: disjointed systems and confusion around whose role it is to provide follow-up care beyond six weeks post-partum. Quantitative findings indicated that the majority of health professionals reported confidence in their own skills to manage women in the antenatal period (62%, 40/79) and slightly lower rates of confidence in the postpartum interval (57%, 33/58).

**Conclusion:**

These findings regarding whose role it is to provide postpartum care, along with opportunities to improve communication pathways and follow up care have informed the design of a complex health intervention to improve health systems and the provision of DIP related care.

## Background

Rates of diabetes in pregnancy (DIP) are high in low and middle-income countries [1], and disproportionate among indigenous women globally [2–5]. In Australia, Aboriginal and Torres Strait Islander women are 1.5 times more likely to have gestational diabetes mellitus (GDM) and 10 times more likely to have pre-existing type 2 diabetes mellitus (T2DM) compared to the general population [6]. Similarly, in Australia’s Northern Territory (NT), rates of GDM and pre-existing T2DM (both referred to as DIP) are higher among Aboriginal than non-Aboriginal women (GDM: 16% vs. 10%; T2DM: 4% vs. < 1%) [7]. In 2015, there were 4009 babies born to 3959 mothers in the NT with 33% of births to Aboriginal women [8].

DIP contributes to several adverse health outcomes for mother and child, including macrosomia, preterm delivery and congenital malformations [9]. GDM also increases the risk of developing T2DM in the future [10]. Optimal care during and following a pregnancy complicated by diabetes provides opportunities for early intervention in the life of the mother and child [11]. In the NT, guidelines are recommended from the Women’s Business Manual, (remote primary care guidelines) [12]. Antenatal care includes: frequent clinical review by a multidisciplinary team, educating women about the impact of elevated glucose levels on their pregnancy, implementing lifestyle measures to reduce glucose levels, and providing medications if glucose levels remain above target despite lifestyle intervention. These guidelines also recommend that measures to recall women for postpartum follow-up are put in place during pregnancy. Post-partum care includes clinical screening to identify women with T2DM (among those with GDM) and providing support for optimal glycaemic levels among women with T2DM [13]. A 75 g Oral Glucose Tolerance Test (OGTT) is recommended 6–8 weeks postpartum for women with a GDM diagnosis [14]. If not possible, a HbA_1c_ at 4 months postpartum is advised [12].

Barriers and facilitators to programs and services addressing DIP have been reported internationally [1]. They have been identified at various levels, including: health systems, individual and social and societal. The remote context of the NT, with a large geographic area and relatively small population, also challenges the delivery of antenatal and postpartum diabetes care in this setting. Care is provided through primary and tertiary health care sectors across the NT. Post-partum care is provided by primary health care and delivered by health care professionals including remote area nurses, Aboriginal health practitioners, remote medical practitioners, midwives, general practitioners and allied health specialists [15]. It is acknowledged that to deliver culturally appropriate care, strategies need to be developed that meet the needs of local contexts [16]. The NT DIP Partnership was established in 2012 and aims to improve health outcomes for women with DIP and their offspring [17]. To date, the work of the Partnership has focused on a health systems approach targeting the antenatal period and identified improved integration in communication and continuity of care [15, 17]. In formative work, health professionals’ reported understanding the importance of preconception care, however the complexity of the care setting and infrequent preconception consultations created challenges [18].

In response to requests from communities and clinicians, the Partnership expanded its focus to improve systems and services in the post-partum period. This mixed-methods study aimed to understand health professionals: (i) perspectives around factors influencing the delivery of antenatal and post-partum diabetes care to women in a high-risk population in the NT and (ii) self-reported confidence of antenatal and post-partum diabetes screening tests and management. The first part of the study required a qualitative methodology, as it aimed to obtain in-depth understandings of factors influencing the delivery of care. This informed the second component where a health professional survey was developed to obtain self-reported information across a broad sample sample of health professionals regarding screening and management.

## Methods

### Participants

Health professionals who participated were from different settings and disciplines, including: selected Primary Health Networks, relevant hospital departments, Aboriginal Community Controlled Health Organisations, and non-Government Organisations. Participants involved in the provision of diabetes screening and management in the pregnancy and postpartum period were eligible (Table 1), and purposefully recruited through the Partnership networks. Invitations to participate in a focus group, interview or online survey were distributed via health professional and stakeholder networks through email, phone call or at relevant educational forums.

**Table 1:** Roles of health professionals

Type of care	Who is involved
DIP Antenatal screening	- primary care doctor, midwife, and sometimes obstetrician (early screening in high risk groups would usually be primary care doctor, as the woman is not usually seen by an obstetirician in first trimester)
DIP Antenatal care	- team-based care, usually involves primary care doctor (GP/Remote Medical Practioner), midwife, obstetrician, diabetes educator, dietitian - possibly endocrinologist depending on needs of woman and/or remote area nurse (or chronic disease nurse if urban)
DIP Post-partum screening	- primary care doctor, midwife and/or nurse
DIP Post-partum care	- immediately post-partum in hospital: midwife, dietitian, diabetes educator, obstetrician, possibly endocrinologist - after discharge: primary care doctor, midwife, diabetes educator, remote area nurse/chronic disease nurse.
Pre-pregnancy counselling	- primary care doctor/nurse (rural health nurse/chronic disease nurse); diabetes educator (if pre-existing); endocrinologist (if referred by GP most women would not see an endocrinologist until pregnant and diagnosed with DIP)

### Qualitative methods

A phenomenological methodology underpinned the qualitative component of this study. Interview questions were informed by previous work of the Partnership. An interview guide was piloted with clinical members of the Partnership and questions adapted over the data collection phase. Data were collected between March and December 2016. Face-to-face data collection was preferred but in cases where geographical distance was too large, data were collected via teleconference. Focus groups were facilitated by RK, CW, PV or CC and interviews by RK. A semi-structured interview guide informed the data collection. With consent, all data were audio recorded and transcribed by NTJ. Transcripts were reviewed by RK and NTJ prior to analysis. One participant did not give permission for their interview to be recorded and notes were taken. In line with a phenomenological approach to data analysis, data were inductively and then deductively analysed in NVivo 11 by RK and NTJ. Interpretations of meaning were cross-checked for accuracy between these two researchers over the data analysis phase (November 2016 and April 2017).

### Quantitative methods

To efficiently capture the perspectives of a broader sample of health professionals, a 37-item survey was developed to obtain insight into health professionals’ perspectives of DIP antenatal and post-partum care (Additional file 1). Survey questions were informed by the qualitative data and constructs from earlier work of the DIP Partnership [14, 15]. The survey was also piloted among members of the Partnership who were clinicians working in this context. Participants were invited to participate between May and November 2017.

Frequencies, percentages and Pearsons’ chi squared were calculated to compare responses according to participant characteristics: health professional group, workplace, setting and years employed in current position. Analyses were undertaken using Stata 15.1 (Stata Corp, US).

## Results

### Qualitative results

Sixty-two health professionals participated in the qualitative component: midwives (37%), diabetes educators (16%), general practitioners (13%), and smaller percentages of nurses, physicians, obstetricians, Aboriginal health practitioners, dietitians and managers. No-one approached by the research team declined the invitation to participate. Time in profession ranged from 6 months to 24 years, 33 worked in remote locations and 29 urban. Seven focus groups were undertaken with 42 participants (five face-to-face; two via phone). Twenty participants participated in 13 interviews (six separately, seven in pairs; 14 face-to-face, five via phone). Duration of interviews and focus groups ranged from 21 to 45 min.

Findings provide insights into factors influencing the delivery of diabetes care, including: roles and responsibilities, communication, context, access to culturally appropriate care and opportunities for improvements (see Table 2 for additional data).

**Table 2:** Additional Qualitative Results

*Additional Themes*	*Supporting Extracts*
Accessing care	
*Barriers to accessing care*	*Sometimes when you go up there for what should be you know a quick five or ten-minute thing […*] *but you might have to wait for a long time. Those kind of factors. I think people get fed up with – you know understandably (Remote Diabetes Educator)*
*Other factors*	*Their relationship with the clinic. And some people don’t feel comfortable in their community clinics. Some – don’t find them comfortable places to be. How busy they are with other things or how many other children they have. Whether they are working. Whether they’re understanding things. The timing of things. (Remote Diabetes Educator)*
	Transience of women living in remote areas also challenges continuity of care in the post-partum period. As a General Practitioner said, ‘*I think the other big problem is that people just drop out of the system.’*
	Many participants described how post-partum diabetes follow up was often opportunistic. Women’s ‘*main reasons for engaging in this service […*] *after they’ve had the baby, it’s mainly [for] the baby – not necessarily the GDM.’*
Pre-conception care	
*Pre-pregnancy planning from a young age*	*Young teenage girls that are diagnosed with Type 2, I am talking with them from the get-go about pre-pregnancy planning. The same as we would for Type 1’s. We’re talking to them from the age of eleven on. We’re starting to talk about pre-pregnancy planning. (Diabetes Educator)*
*Whose responsibility is it?*	*Who is it that spends most time talking to women about pregnancy and babies? They’re the ones that need to talk about it.’ (Remote Diabetes Educator)*
*Reported understanding of the importance of contraception & glucose control*	*I’ve actually had a couple [of women] in a couple of communities that have come back out to me saying “I want to get my Implanon out, but I need to see you first. I need to talk to you about where my sugars have got to be.” (Diabetes Educator)*
*Reported increased awareness among some women*	*… other women in the community have noticed, and I’ve actually had a few from [that community] that have actually come in [to clinic] wanting to do the pre-pregnancy planning – because they don’t want what happened to her to happen to them. (Diabetes Educator)*

#### The challenges of an OGTT

Many health professionals described how post-partum OGTT follow-up was commonly missed. One explanation related to a lack of clarity around whose responsibility it is:

*The Child Health Nurse will see [a recall for a post-partum OGTT] and think the Midwife will do it, and the Midwife will see it and think the Child Health Nurse will do it. Or the Doctor will see it and think, oh, one of the Nurses will do it.*

Participant 1, Remote Diabetes Educator

Other reasons included staff turn-over and transience: *‘A lot of the people here are transient. A lot of the doctors are transient. So, it makes it a very challenging system to work within.’*

Participant 2, Diabetes Educator

A common discourse, as clearly articulated by Participant 3 (a Remote Midwife) is that *‘our biggest problem is getting the women fasting and staying in the clinic for two hours’.* As also described by Participant 4 (a Remote Outreach Midwife), further compounding this was *‘women dislike the test [and] hanging around for two hours.’*

#### Disjointed systems and communication

Variation in timeliness and quality of discharge summaries was also described as problematic. Participant 5 (an Outreach Dietician) said they *‘often don’t exist or come weeks later’.* Participant 3 (a remote midwife) reinforced this variability: *‘we sometimes get a phone call [from the hospital] advising us they are coming home. We sometimes get nothing.’*

Consequently, Participant 1 (a Remote Diabetes Educator) explained that:

*… there’d be quite a few women out there who had GDM or DIP and have probably not had a postpartum check to see whether they still have diabetes.*

This was supported by others, including Participant 6 (Diabetes Educator) who had *‘picked up a couple’* of women ‘*in communities where they’ve come in with their babies that are six or seven months old [ …*] *you’ll do a random sugar and they’ve got blood sugars of 20 because they’ve stopped the treatment that they were having while they were pregnant.’*

The need to deliver care that meets the needs of women and is culturally appropriate was also raised. Participant 1 (Remote Diabetes Educator) summarized that *‘to improve services we need to have more Aboriginal people employed looking after Aboriginal health.’*

#### The baby is the focus

There was consensus among many of the health professionals interviewed, that following the pregnancy, *‘mums are willing to come in for an appointment for their children, but then we struggle [ … ] to get our mums to present for themselves.’*

Participant 3, Remote Midwife.

One strategy to overcoming missed post-partum follow up and providing continuity of care to women in this period is to:

*Hav [e] a dedicated nurse whose role is to care for the zero to five-year olds. Including [ … ] the postnatal [clinical care of] mothers up to the six-week period [ … ] and [someone who] will bring them into the clinic for the follow-up appointments in relation to ongoing medical issues.*

Participant 3, Remote Midwife.

#### Pre-conception care

The post-partum period was recognized as an opportune time to promote preconception health. Although variability in this practice was raised, Participant 1 (a Remote Diabetes Educator) stated:

*… we’re particularly careful that we make sure we talk to [young women with T2DM] about pre-conception counselling and pregnancy and having the diabetes under control before it happens [ … ] quite often women don’t actually come to the clinic [ … ] if [they]‘re feeling well [ … ]so [they] don’t actually go to the clinic for anything until [they]‘re pregnant.*

### Quantitative results: health professional survey

The survey was completed by 82 health professionals, with a similar mix from Central Australia (*n* = 36) and the Top End (*n* = 46) (Table 3). These two regions provide models of care relevant to their context and were analysed separately to review any differences. Of all participants, 57% had been in their current position for 0–5 years, 17% for 5–10 years and 26% for more than 10 years. Table 4 outlines survey responses according to health professional characteristics.

**Table 3:** Health Professional Survey: respondent demographics

Characteristic	Frequency (%)
Main work setting (*n* = 82)	
Top End Regional or Remote	17 (20)
Top End Urban	28 (34)
Central Australia Regional or Remote	20 (24)
Central Australia Urban	17 (21)
Time in position (*n* = 82)	
1 year	23 (28)
1–5 years	24 (29)
5–10 years	14 (17)
> 10 years	21 (26)
Primary work place (*n* = 75)	
General Practice	11 (15)
Health Centre	32 (43)
Hospital	13 (17)
Other	19 (25)
Client base (*n* = 78)	
Aboriginal or Torres Strait Islander women	48 (62)
Non Aboriginal or Torres Strait Islander Women	8 (10)
Mixed	22 (28)
What percentage of women with DIP have you seen for pre-pregnancy counselling? (*n* = 55)	
0–20%	43 (78)
20–40%	5 (9)
> 40%	7 (14)
What percentage of women would you also see post-partum? (*n* = 56)	
0–20%	20 (36)
20–40%	12 (21)
> 40%	24 (43)

**Table 4:** Health Professional opinions of current practice according to participant characteristics

	Health professional					Region			Location			Time in role (years)			Total
	Med Pract *n* = 23	Nurse *n* = 25	Dietician/DE *n* = 20	Other *n* = 14	*p*	CA	Top End	p	Urban	Remote	*p*	0–5	5+	*p*	
Screening															
Antenatal screening															*n = 76*
*Yes*	17 (81)	19 (76)	7 (39)	5 (42)		23 (66)	25 (61)		22 (73)	26 (57)		21 (49)	27 (82)		48 (63)
*No/unsure*	4 (19)	6 (24)	11 (61)	7 (59)	0.009	12 (34)	16 (39)	0.67	8 (27)	20 (43)	0.14	22 (51)	6 (18)	0.003	28 (37)
Postpartum screening															*n = 54*
*Yes*	16 (94)	17 (89)	3 (27)	4 (57)		19 (76)	21 (72)		18 (86)	22 (67)		17 (61)	23 (88)		40 (74)
*No/unsure*	1 (6)	2 (11)	8 (73)	3 (43)	< 0.001	6 (24)	8 (28)	0.76	3 (14)	11 (33)	0.12	11 (39)	3 (12)	0.02	14 (26)
When screening**															*n = 49*
*At 6 weeks*	9 (53)	12 (71)	3 (30)	2 (40)		13 (54)	12 (52)		14 (74)	12 (40)		14 (56)	12 (50)		26 (53)
*From 3 to 12 months*	8 (47)	5 (29)	7 (70)	3 (60)	0.21	11 (46)	12 (48)	0.88	5 (26)	18 (60)	0.021	11 (44)	12 (50)	0.67	23 (47)
Pre-pregnancy counselling:															*n = 55*
*≤ 20%*	12 (67)	17 (89)	9 (82)	5 (71)		21 (84)	22 (73)		14 (70)	29 (83)		25 (86)	18 (69)		43 (78)
*> 20%*	6 (33)	2 (11)	2 (18)	2 (29)	0.38	4 (16)	8 (27)	0.34	6 (30)	6 (17)	0.27	4 (14)	8 (31)	0.13	12 (22)
Confidence															
Antenatal management															*n = 79*
*Confident*	15 (71)	15 (40)	14 (74)	5 (36)		19 (54)	30 (68)		18 (58)	31 (65)		27 (59)	22 (67)		49 (62)
*Not confident*	10 (40)	6 (29)	5 (26)	9 (64)	0.11	16 (46)	14 (32)	0.21	13 (42)	17 (35)	0.56	19 (41)	11 (33)	0.47	30 (38)
Post-partum interval															*n = 58*
*Confident*	14 (78)	10 (56)	6 (46)	3 (33)		15 (60)	18 (55)		11 (52)	22 (59)		18 (55)	15 (60)		33 (57)
*Not confident*	4 (22)	8 (44)	7 (54)	6 (67)	0.12	10 (40)	15 (45)	0.68	10 (48)	15 (41)	0.60	15 (45)	10 (40)	0.68	25 (43)
Pre-pregnancy counselling															*n = 55*
*Confident*	13 (76)	11 (58)	8 (73)	4 (50)		16 (64)	20 (67)		10 (53)	26 (72)		20 (69)	16 (62)		36 (65)
*Not confident*	4 (24)	8 (42)	3 (27)	4 (50)	0.48	9 (36)	10 (33)	0.84	9 (47)	10 (28)	0.15	9 (31)	10 (38)	0.56	19 (35)

This was a self-report survey with measures such as *appropriate*, *screening* and *confidence* all reflecting health professionals views. Screening in the post-partum period refers to screening those without pre-existing diabetes for type 2 diabetes

*Medical Practitioner includes General Practitioner and Physician/Obstetrician; Nurses include midwives and registered nurses; DE = Diabetes Educator; Other includes AHP and Managers

**This screening refers to HbA1c testing in T2DM in the post-partum period

Quantitative findings provided further insight into factors influencing screening and self-reported confidence of health professionals. Where relevant, findings have been interpreted in line with insights obtained from the qualitative component of the study. No significant differences were found when comparing responses by regions.

#### Antenatal and post-partum screening

A high proportion 48/76 of survey respondents reported routinely screening all women without pre-existing diabetes in the antenatal period, and 40/54 reported screening women after GDM. Diabetes educators and dieticians, compared to nurses and medical practitioners, along with those being in their role for < 5 compared to > = 5 years had significantly lower rates of routine antenatal or postpartum screening.

For women with pre-existing diabetes, HbA_1c_ testing was more likely reported as recommencing at 6 weeks post-partum by those in urban centers and after 3 months by those in remote locations. Post-partum screening of women who had GDM was reported as occurring opportunistically (12%, *n* = 6/50), by recall (32%, *n* = 16/50) and both (56%, *n* = 28/50).

**Fig. 1:**
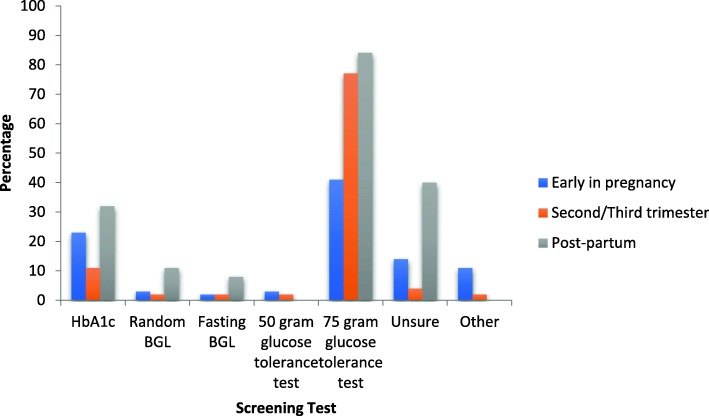
Health professionals self-reported use of screening tests

The 75-g oral glucose tolerance test was reported as being the most appropriate in all periods. Thirty four percent (20/58) reported that they usually have access to measure HbA_1c_ at the point of care (Fig. 1).

#### Confidence

Sixty two percent (49/79) of respondents reported confidence in their own skills to manage women in the antenatal period and 57 % (33/58) in the post-partum interval. Of those who were not confident in the post-partum period, 64% did not routinely screen women with a history of GDM for impaired glucose tolerance or T2DM. Confidence in explaining the associated risks of insulin use for breastfeeding women was low (24%, *n* = 14). Sixty-five percent (36/55) reported confidence in managing pre-pregnancy counselling for women who had a history of pre-existing T2DM or GDM.

## Discussion

This study reports three main findings related to the delivery of ante-natal and post-partum diabetes care in Australia’s NT. Firstly, disjointed systems and communication pathways created challenges for providing timely clinical follow-up that is culturally appropriate. This was further complicated by the remote context. Secondly, only 57% of health professional’s reported confidence in screening and delivering care in the post-partum period. Thirdly, opportunities to improve care for women at risk of diabetes following pregnancy were identified, including: enhanced education and support of health professionals, improved communication pathways and strengthening the Aboriginal workforce.

The qualitative data highlighted health systems and individual challenges in delivering post-partum care for women who had DIP, echoing a recent systematic review [1]. This included limitations with communication pathways, such as inconsistencies with discharge summaries not being sent to relevant primary health care centres, consistent with a review of maternal and infant health services in the region [19], along with international literature [1, 20]. Furthermore, clarity around whose role it was to administer diabetes screening tests and provide follow-up care in the post-partum period was limited. Siloed approaches to the provision of care were evident, leading to potentially compromised continuity of care and poor post-partum follow-up with opportunities for diabetes screening missed. Our findings are consistent with others, highlighting that for women who had GDM, providing an OGTT in the post-partum period was challenging [16, 21, 22] and that women often prioritise the needs of their family over their own post-partum health [16]. Other patient-related barriers to accessing diabetes post-partum care include women not perceiving the future risk of developing T2DM, having limited motivation to maintain healthy behaviours after the birth, time constraints and the cost of transport [22]. Good client-provider relationships are also important facilitators for screening and treatment [23]. Health professionals suggested the post-partum period is an opportune time to undertake relevant diabetes related health checks, an approach also recommended by the international federation of gynaecology and obstetrics [24].

Challenges in delivery of culturally-appropriate care were also evident. Contributors include disjointed systems and communication pathways, along with high staff turnover and its negative impact on continuity of care. Social and cultural factors have implications for the uptake of health care programs, which should be designed and implemented accounting for context [1]. Specifically, supportive environments are critical to diabetes prevention [25]. It is well understood that Aboriginal women value continuity of maternal care and the involvement of an Aboriginal health workforce in the delivery of this care [16]. However, the current Aboriginal health workforce in the NT is small and significant strengthening in numbers of this workforce is required in order to deliver care required across this large jurisdiction [26]. Health care services should involve partnerships with Aboriginal and Torres Strait Islander families, community and workers [27], and be responsive to the needs of Aboriginal people [28, 29].

In the survey, only 57% of health professionals reported confidence in delivering diabetes care in the post-partum period. As highlighted by the qualitative findings, this could relate to follow-up of women with GDM being perceived as the role of other health professionals (i.e. midwives) or associated with the emphasis on monitoring women during pregnancy when compared to after the birth. Furthermore, low confidence in explaining risk of breastfeeding for women on insulin was reported. Those working in rural and remote locations tended to report greater confidence in providing pre-pregnancy counselling, particularly for those who had a history of pre-existing diabetes or GDM, when compared to urban. This may be related to the opportunity of clients and health professionals to establish long-term relationships in rural and remote settings [30], which was also described in the qualitative results. Overall, rates for pre-conception counselling were low, echoing findings from a recent NT study exploring preconception care for women with T2DM [18]. As reported elsewhere, low presentation rates to Primary Health Care clinics in the preconception period may influence pre-conception counselling [18, 31, 32].

This study identified opportunities to improve antenatal and postpartum care for women with DIP. It was evident that enhancing education and support of health professionals to screen and provide post-partum clinical care would be beneficial. Successful models for enhancing the capacity of the health workforce in this remote context include establishing networks and providing education [15, 17]. Improving communication pathways and integration of relevant health systems is also important and has been found to overcome issues caused from disjointed referral pathways, discharge summaries and follow up of women. Clinical registers are a tool that have been reported to improve such pathways [33, 34]. Additionally, strengthening the Aboriginal health workforce is a strategy to improve access to culturally appropriate care [16].

### Strengths and limitations

The study findings are based on health professionals’ perceptions of DIP screening and management, providing expert opinion around service delivery in this context. It is likely that these findings are also of relevance to other regions with similar contexts and possibly to a broader range of health care contexts. Participation was voluntary and it is unknown how many health professionals were invited to participate in the survey (nor what the survey response rate was). Of participants who did not respond to all survey questions, most skipped the same questions suggesting a possible lack of relevance to their role (e.g. provide antenatal care only). Another major limitation was not including clients of health services to comment on their experiences of diabetes related health care. This was beyond the scope of this study, but is currently being addressed by other work of the Partnership. Strengths of the study are the inclusion of a broad range of health professionals across a large jurisdiction. It is the first study exploring health professionals’ perspectives around the delivery of post-partum diabetes care in the context of a remote region with a high-risk population. It highlights opportunities to enhance care in the post-partum interval in remote settings for high risk women, which are likely of global relevance. An audit to assess current practice will also be performed by the Partnership.

### Implications for practice

These findings have informed the Partnership’s development of a multi-component complex health systems intervention across the NT and Far North Queensland, and the development of auditing methods to inform best practice. Key components of the intervention include: (i) improving information management and communication, (ii) improving access to culturally and clinically appropriate care, (iii) increasing workforce capacity and the health literacy of health professionals and women in postpartum diabetes care, (iv) using the DIP clinical register for continuous quality improvement activities, and (v) enhancing policy and guidelines.

## Conclusion

In conclusion, this study provides insights into the strengths and barriers of providing post-partum management of women after DIP in remote northern Australia, which echo those in international settings. As outlined, ongoing work of the Partnership seeks to address some of these challenges and improve health outcomes for at risk populations in this context.

## Supplementary information

**Additional file 1.** Survey, Survey of health care professionals in the NT.

## Data Availability

The data generated and analysed during the current study are not publicly available. Researchers may apply for access to these data through The NT DIP Partnership Editorial Committee by contacting the Principal Chief Investigators of the NT DIP Partnership, Professor Louise Maple-Brown (email: louise.maple-brown@menzies.edu.au).

## Abbreviations

DIP: Diabetes in Pregnancy
GDM: Gestational Diabetes Mellitus
NT: Northern Territory
OGTT: Oral Glucose Tolerance Test
T2DM: Type 2 Diabetes Mellitus
