# Supplementary material for: Diabetes care in remote Australia: the antenatal, postpartum and inter-pregnancy period
Source: BMC Pregnancy Childbirth. 2019 Oct 28;19:389. doi: 10.1186/s12884-019-2562-6 (PMC6819653; doi:10.1186/s12884-019-2562-6)
Supplement: Supplementary file 1 — Additional file 1. Survey, Survey of health care professionals in the NT. [file 12884_2019_2562_MOESM1_ESM.pdf]

# NT Diabetes in Pregnancy
## Survey for health care professionals in the NT
May 2017

## Consent

**This survey is for Health Professionals involved in the management of women with diabetes before, during or after pregnancy. The survey examines attitudes and knowledge of all aspects of the care of pregnant women with diabetes. It aims to identify gaps between current evidence and practice in management as well as determine key areas for improvement in the current models of care. Results will be reported grouped and de-identified to relevant professional and scientific bodies. In order to ensure security of the data, survey results will be downloaded and then deleted from the online electronic survey www.surveymonkey.com site and stored in a secure computer within Menzies School of Health facilities.**

**If you have questions about the survey please contact Menzies School of Health NT & FNQ Diabetes in Pregnancy Partnership Coordinator Cherie Whitbread on 89468696 or email Cherie.whitbread@menzies.edu.au. If you have any concerns or complaints about the survey or project, you may contact the Secretary of the Human Research Ethics Committee of NT Department of Health and Menzies School of Health Research Phone 08 89468687 or ethics@menzies.edu.au**

1. Please indicate your consent in order to proceed with the survey:

- ◯ I agree
- ◯ I do not agree

2. Are you a primary health care worker?

- ◯ Yes
- ◯ No

NT Diabetes in Pregnancy
Survey for health care professionals in the NT
May 2017

## Individual context

**The following questions collect some information about you.**

3. What is your current occupation? Choose one

- ○ Registered Nurse
- ○ Registered Midwife
- ○ General Practitioner
- ○ Medical Specialist
- ○ Medical Practitioner (other, please specify below)
- ○ Diabetes Educator
- ○ Aboriginal and Torres Strait Islander Health Practitioner
- ○ Manager
- ○ Dietician
- ○ other ( please specify below)

Other please specify

[ ]

4. How long have you been in your current position?

- ○ 1 year
- ○ 1-5 years
- ○ 5-10 years
- ○ >10 years

5. In which region of the NT do you work?

- ☐ Central urban
- ☐ Central regional or remote
- ☐ Top End, urban
- ☐ Top End, regional or remote

6. What is your primary work place?

- ◯ General practice
- ◯ Health centre
- ◯ Hospital
- ◯ Other (please specify)

## Your Clinical Practice

**The following questions aim to collect some information about your clinical practice when working with women who have diabetes before, during and after pregnancy.**

7. Are the clients you work with predominantly?

- ◯ Aboriginal or Torres Strait Islander
- ◯ Non-Aboriginal or Torres Strait Islander
- ◯ Mixed

8. How confident are you in your own skills to manage women with diabetes in pregnancy?

- ◯ Not at all confident
- ◯ Not confident
- ◯ Neutral
- ◯ Confident
- ◯ Very confident

9. Do you believe most women in your health service or hospital receive appropriate care for diabetes during pregnancy.

- ◯ Strongly disagree
- ◯ Disagree
- ◯ Neutral
- ◯ Agree
- ◯ Strongly Agree

10. Do you believe most women in your health service or hospital receive appropriate care for diabetes after pregnancy.

- ◯ Strongly disagree
- ◯ Disagree
- ◯ Neutral
- ◯ Agree
- ◯ Strongly Agree

11. Do you routinely screen any women for diabetes early in pregnancy (at first visit or soon after)?

- ◯ No
- ◯ Yes
- ◯ Unsure

## Your clinical practice

12. If yes, who do you usually screen for diabetes early in pregnancy (at first visit or soon after) tick all that apply

- [ ] Everyone
- [ ] All Aboriginal women
- [ ] Other higher risk ethnic groups
- [ ] Older maternal age (>35)
- [ ] Obese
- [ ] Family history of diabetes or gestational diabetes
- [ ] Personal history of abnormal glucose tolerance
- [ ] Previous pregnancy complications
- [ ] Previous large baby
- [ ] Glycosuria
- [ ] Polycystic ovary syndrome
- [ ] Hypertension or pregnancy-induced hypertension
- [ ] Use of glucocorticoids (e.g. prednisolone)
- [ ] As per electronically generated list of antenatal bloods
- [ ] Unsure

13. What screening test do you use most commonly for gestational diabetes early in pregnancy (or at first attendance to a health centre)?

- ◯ HbA1c
- ◯ Random BGL
- ◯ Fasting  BGL
- ◯ 50 gram glucose challenge test(GCT)
- ◯ 75 gram glucose challenge test (GTT)
- ◯ unsure
- ◯ Other (please specify)

14. Do you routinely screen any women for diabetes early in thesecond or third trimester?

- ◯ No
- ◯ Yes
- ◯ Unsure

## Your Clinical Practice

15. If yes, who do you usually screen for diabetes in the second or third trimester? Tick all that apply.

- ☐ Everyone
- ☐ All Aboriginal women
- ☐ Other higher risk ethnic groups
- ☐ Older maternal age (>35)
- ☐ Obese
- ☐ Family history of diabetes or gestational diabetes
- ☐ Personal history of abnormal glucose tolerance
- ☐ Previous pregnancy complications
- ☐ Previous large baby
- ☐ Glycosuria
- ☐ Polycystic ovary syndrome
- ☐ Hypertension or pregnancy-induced hypertension
- ☐ Use of glucocorticoids (e.g. prednisolone)
- ☐ As per electronically generated list of antenatal bloods
- ☐ Unsure

16. At what gestation do you aim to screen for diabetes in the second or third trimester?

- ○ 24-28 weeks
- ○ 26-28 weeks
- ○ 28 weeks
- ○ <24 weeks
- ○ >28 weeks

17. What screening test do you use most commonly use in the second or third trimester?

- ◯ HbA1c
- ◯ Random BGL
- ◯ Fasting BGL
- ◯ 50 gram glucose challenge test(GCT)
- ◯ 75 gram glucose challenge test (GTT)
- ◯ unsure
- ◯ Other (please specify)

18. Do you routinely screen all women for diabetesafter gestational diabetes in pregnancy?

- ◯ No
- ◯ Yes
- ◯ Unsure

19. How do you usually screen women after they have had gestational diabetes in pregnancy?

- ◯ Opportunistically
- ◯ Recall
- ◯ Both

20. When do you usually screen women after they have had gestational diabetes in pregnancy?

- ◯ At 6 weeks
- ◯ Up to 12 weeks
- ◯ 13 weeks - 6 months
- ◯ 6-12 months

21. What screening test do you use most commonly for testing for diabetes after gestational diabetes? Please rank the top 3 in order of your preferred test.

- [ ] HbA1c
- [ ] Random BGL
- [ ] Fasting  BGL
- [ ] 50 gram glucose challenge test(GCT)
- [ ] 75 gram glucose challenge test (GTT)
- [ ] unsure

22. After the first screening test, when the result was within normal range, how often do you routinely screen women for diabetes who have had gestational diabetes?

- ○ Before next pregnancy/stopping contraception
- ○ Yearly
- ○ 2nd yearly
- ○ Other (please specify)

23. Approximately what percentage of the women with diabetes in pregnancy that you work with have you seen prior to their pregnancy specifically for pre-pregnancy counselling?

- ○ 0-20%
- ○ 20-40%
- ○ 40-60%
- ○ 60-80%
- ○ 80-100%

24. How confident are you in your own skills to manage women with a history of pre-existing or gestational diabetes in pregnancy to manage pre-pregnancy counselling?

- ◯ Not at all confident
- ◯ Not confident
- ◯ Neutral
- ◯ Confident
- ◯ Very confident

25. Approximately what percentage of pregnant women that you work with do you see post-partum for ongoing clinical care (eg 6 week check)

- ◯ 0-20%
- ◯ 20-40%
- ◯ 40-60%
- ◯ 60-80%
- ◯ 80-100%

26. How confident are you in your own skills regarding diabetes management for women with diabetes in pregnancy in the post-partum interval?

- ◯ Not at all confident
- ◯ Not confident
- ◯ Neutral
- ◯ Confident
- ◯ Very confident

27. For women with pre-existing diabetes, at what time do you recommence HbA1c testing after pregnancy?

- ◯ 6 weeks
- ◯ 3 months
- ◯ 6 months
- ◯ 12 months

28. What management would you be most likely to suggest for women with pre-existing diabetes who are breastfeeding, prescribed metformin 2G daily and have a HbA1c of between 7 – 8.5%?

- ◯ Lifestyle changes (nutrition, exercise, smoking cessation), continue contraception and review in 3 months.
- ◯ Lifestyle changes (nutrition, exercise, smoking cessation), continue contraception, blood glucose monitoring and review in 2 weeks.
- ◯ Lifestyle changes (nutrition, exercise, smoking cessation), continue contraception, blood glucose monitoring, and another oral or injectable diabetes agent and review in 2 weeks.
- ◯ Other (please specify)

29. How confident are you explaining the associated risks for breastfeeding women who are prescribed insulin?

- ◯ Not at all confident
- ◯ Not confident
- ◯ Neutral
- ◯ Confident
- ◯ Very confident

30. Do you usually have access to a point of care (on site) HbA1c tester?

- ◯ Yes
- ◯ No
- ◯ Unsure

## Your clinical practice

31. If yes how does that access impact your use of HbA1c in the management of pregnant women?

- ○ Nil
- ○ test more frequently
- ○ test less frequently
- ○ Other (please specify)

32. What professional educational resources do you use on a regular basis? tick all that apply

- ☐ Medical databases (e.g. PubMed, Medline)
- ☐ Carpa
- ☐ Women's Business Manual (including Reference Manual)
- ☐ Prompt guidelines
- ☐ UpToDate
- ☐ Rural Health Education Foundation
- ☐ Other specialist websites (e.g. South Australia Perinatal Guidelines)
- ☐ Australian Diabetes in Pregnancy Society (ADIPS) guidelines
- ☐ NH&MRC antenatal guidelines
- ☐ Other (please specify)

33. Please rank the following from 1 – 5 in order of preference for professional education about Diabetes in Pregnancy

- [ ] Course
- [ ] Lecture or talk by specialist
- [ ] Online modules
- [ ] Personal learning (e.g. journal articles, textbook)
- [ ] As part of a conference or symposium

34. For client education about diabetes in pregnancy, which pamphlets, brochures or flip-charts do you use? tick all that apply

- [ ] Healthy Living NT flipchart or poster
- [ ] Queensland Department of Health resources
- [ ] Royal Darwin Hospital resources
- [ ] Australian Diabetes in Pregnancy Society (ADIPS)
- [ ] Other (please specify)

35. For client education about diabetes before or after pregnancy, which pamphlets, brochures or flip-charts do you use? tick all that apply

- [ ] Healthy Living NT flipchart or poster
- [ ] Queensland Department of Health resources
- [ ] Royal Darwin Hospital resources
- [ ] Australian Diabetes in Pregnancy Society (ADIPS)
- [ ] Other (please specify)

36. Have you been involved in telephone or video case conferences for any client condition in your health service or hospital?

- ( ) Yes
- ( ) No

## Your clinical practice

37. If yes, how useful have you found telephone or video case conferencing as part of client care?

- Not at all
- Not useful
- Neutral
- Useful
- Very useful
